# Quantitative analysis of APC promoter methylation in hepatocellular carcinoma and its prognostic implications

**DOI:** 10.3892/ol.2014.1951

**Published:** 2014-03-07

**Authors:** BAIYING XU, YANFANG NIE, XIAOXIA LIU, SHUQIN FENG, ZHILI YANG, ZHIGANG WANG, QI ZHENG, XIAOYING LUO

**Affiliations:** 1 Department of General Surgery, Shanghai No. 6 People’s Hospital, Medical School of Shanghai Jiaotong University, Shanghai, P.R. China; 2 Department of Nephrology, Taizhou Central Hospital, Taizhou, Zhejiang, P.R. China; 3 The Obstetrics and Gynecology Hospital, Fudan University, Shanghai, P.R. China; 4 Shanxi Province Industry and Trade College, Taiyuan, Shanxi, P.R. China; 5 State Key Laboratory of Oncogenes and Related Genes, Shanghai Cancer Institute, Renji Hospital, Medical School of Shanghai Jiaotong University, Shanghai, P.R. China

**Keywords:** DNA methylation, methylight, hepatocellular carcinoma, prognostic biomarker, APC

## Abstract

The present study aimed to quantitatively determine the aberrant methylation signal of the adenomatous polyposis coli (APC) gene in hepatocellular carcinoma (HCC), and to evaluate whether hypermethylation of the APC promoter could be a prognostic biomarker for HCC. Taqman probe-based quantitative methylation-specific polymerase chain reaction was performed to identify the APC promoter methylation levels in 57 HCC and corresponding non-tumorous liver tissues. In the present study, the methylation level of the APC promoter was upregulated by 4.51-fold in the HCC tissues compared with the non-cancerous tissues (P=0.0003). With regard to the clinicopathological data, the methylation level of the APC promoter in the HCC samples was higher in the patients with larger tumors when the cut-off was set at 4 cm (P=0.0008), and in the older patients when the cut-off was set at 60 years old (P=0.0438). However, the methylation status in the HCC samples appeared not to affect the overall patient survival rate (P=0.1684). The findings of the present study showed that APC promoter hypermethylation accumulates during the development of HCC, but that it may not be a promising prognostic biomarker for HCC.

## Introduction

Hepatocellular carcinoma (HCC) is the leading cause of cancer-related mortality worldwide, particularly in Asia. Significant risk factors include hepatitis C virus infection, hepatitis B virus infection and cirrhosis. Previously, numerous genetic and epigenetic alterations have been associated with hepatocarcinogenesis (1). Hypermethylation of gene promoters can be promising tools, for example as biomarkers, in the detection of cancer cells in tissues and body fluids. The clinical value of methylation markers has been reported for the early detection and classification of cancer, for risk assessment and prognosis and for the prediction of therapy response. The ability to use methylation markers for diagnostics and as a predictive tool for cancer is becoming tangible (2).

The adenomatous polyposis coli (APC) tumor suppressor gene encodes a large protein with multiple cellular functions and interactions, including signal transduction in the Wnt-signaling pathway (3). Defects in this gene cause familial adenomatous polyposis. Numerous studies have shown that the inactivation of APC by promoter hypermethylation is frequent in HCC. To the best of our knowledge, previous studies have used different detection methods and therefore the reported methylation frequencies/levels of the APC promoter in HCC have varied among the studies. Using the same method has also produced varied methylation levels/frequencies (2,4–12). Furthermore, in these studies the methylation signal in the APC promoter was determined by a qualitative or quantitative assay in ≤50 paired samples.

The present study aimed to obtain a quantitative methylation signal of APC in HCC, using the more exact quantitative method of MethyLight, which is based on Taqman probes, to detect the promoter of the APC gene in 57 paired HCC and matched non-malignant liver tissues. The correlation between the methylation status and clinicopathological features was analyzed further, and the ability of APC to serve as a potential biomarker of the prognosis for HCC was evaluated.

## Materials and methods

### Human tissues

Human primary HCC and corresponding non-cancerous liver tissues (3 cm from the tumor) were collected from 57 patients who were diagnosed and treated at Guangxi Medical University, Guangxi, China, between January 2003 and June 2005. The study protocol was approved by the Clinical Research Ethics Committee of Shanghai Cancer Institute (Shangai, China) and informed consent was obtained from each patient. The tissue samples were snap-frozen in liquid nitrogen immediately after surgical resection and then stored at −80°C until analysis. Clinical information was collected from patient records and pathology reports. All patients were followed up from the date of surgery and the survival and mortality rates were recorded. Clinical information is recorded in Table I.

### DNA preparation

Total genomic DNA was extracted from frozen tissue specimens (50–100 mg) according to the standard protocol, with specific modifications, which are briefly described as follows. Frozen pulverized powders of the specimens were resuspended with 2 ml warmed lysis buffer: [50 mM Tris-HCl (pH 8.0), 50 mM EDTA, 1% SDS, 10 mM NaCl and 100 μg/ml boiling-treated RNase A; Sigma-Aldrich, St. Louis, MO, USA]. Following a 1-h incubation at 37°C, proteinase K (Roche Diagnostics, Indianapolis, IN, USA) was added to the cellular lysates to form a 100 μg/ml final concentration, and the digestion was carried out at 55°C for 2 h. Organic extractions with a half volume of phenol/chloroform/isoamyl alcohol (1:1:0.04) were repeatedly performed until no visible interphase remained following centrifugation at 12,000 × g for 10 min. DNA was precipitated from the aqueous phase in the presence of 0.3 M NaOAc (pH 7.0); and two and one-half volumes of ethanol. The DNA pellet was washed once with 70% ethanol and dissolved at 65°C for 30 min with 0.2–0.4 ml Tris-EDTA (TE) buffer [10 mM TrisHCl (pH 7.4) and 1 mM EDTA], followed by storage at 4°C until further use. The DNA concentrations were calculated according to their optical density readings at 260 nm (13).

### CpG methyltransferase (M.SssI) methylation assay

Peripheral blood leukocyte (PBL) DNA (Promega Corporation, Madison, WI, USA) was used as a substrate for the M.SssI treatment. PBL DNA (0.05 μg/μl) was incubated with M.SssI at a concentration of 1 U/μg DNA (0.05 U/μl) and 0.16 mM AdoMet overnight at 37°C. Extra AdoMet (to 0.20 mM) and M.SssI (to 0.065 U/μl) were then added followed by a second overnight incubation at 37°C. The sample was stored at 4°C and 18-μl (0.9 μg DNA) aliquots were used for the bisulfite conversion and recovery (14).

### Bisulfite treatment

DNA (10 μg in 50 μl TE) was incubated with 5.5 μl 3 M NaOH at 37°C for 10 min, followed by a 16-h treatment at 50°C, subsequent to the addition of 30 μl freshly prepared 10 mM hydroquinone and 520 μl freshly prepared 3.6 M sodium-bisulfite (pH 5.0). DNA was desalted using a home dialysis system with 1% agarose (detailed protocol will be provided upon request). The DNA in the desalted sample (~100 μl) was denatured at 37°C for 15 min with 5.5 μl 3 M NaOH followed by ethanol precipitation with 33 μl 10 M NH_4_OAC and 300 μl ethanol. Subsequent to washing with 70% ethanol, the gently dried DNA pellet was dissolved with 30 μl TE at 65°C for 10 min. The DNA sample was finally stored at −20°C until further use. A 50-ng DNA sample was reserved for polymerase chain reaction (PCR) (15).

### MethyLight (Taqman probe-based quantitative methylation-specific PCR)

The PCR was performed using a 96-well optical tray with caps at a final reaction volume of 20 μl. Samples contained 8 μl Real MasterMix (Taqman; Tiangen Biotech Co., Ltd., Beijing, China), 1 μl bisulfite-treated DNA, 250 nM each primer and 125 nM 6-carboxyfluorescein-labeled probes. The modified DNA was amplified by MethyLight quantitative PCR (qPCR) using the TaqMan gene assay and the 7500/7500 Fast Real-Time PCR System (Applied Biosystems, Foster City, CA, USA). Primers and probes for APC and Alu-C4 were as previously described (4,14). Each PCR program consisted of an initial denaturation cycle (95°C for 10 min), 45 cycles of denaturation (95°C for 15 sec) and finally an annealing/extension cycle (60°C for 1 min). The methylation ratio was determined by absolute quantification of qPCR. The quantity of amplified target genes in the test samples was normalized with that of Alu-C4 to measure the levels of input DNA, and the DNA treated with M.SssI served as a methylated reference. The amount of methylated DNA, or the percentage of methylated reference (PMR), at a specific locus was calculated by dividing the gene:Alu-C4 ratio of a sample by the gene:Alu-C4 ratio of the M.SssI-treated human genomic DNA (presumably fully methylated) and multiplying by 100.

### Statistical analysis

Data are expressed as the mean ± standard error of the mean from at least three separate experiments. The data were analyzed with either a two-tailed Student’s t-test/χ^2^ test or a one-way analysis of variance for the comparison of more than two groups unless otherwise specified. Kaplan-Meier (KM) method and log-rank test were used to derive the overall survival function, and the log-rank test was used to compare the curves for the two groups. For KM analysis, the median PMR level was used as a cut-off level. Therefore, the definition varied for each gene with the aim of obtaining equal sample sizes for each KM curve. This provided an improved power to identify whether there is an association between the level of methylation in HCC tissues and overall patient survival rate. P<0.05 was considered to indicate a statistically significant difference.

## Results

### Methylation level of the APC promoter in HCC and corresponding non-cancerous tissues

**Figure 1:**
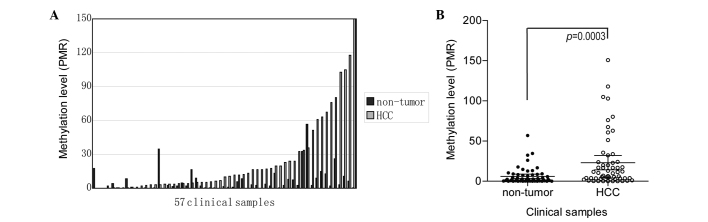
Methylation level of the APC gene promoter in the HCC samples is higher than in the non-tumor liver tissues. (A). Taqman probe-based quantitative methylation-specific PCR (MethyLight) results showing the methylation level of the APC promoter in 57 HCC and matched non-tumor liver tissues. The level of methylation was evaluated by the percentage of methylation (PMR). (B) The methylation level of the APC promoter in the HCC samples was significantly higher than in the paired non-tumor liver tissues. HCC, hepatocellular carcinoma; APC, adenomatous polyposis coli; PCR, polymerase chain reaction.

In order to obtain the methylation signal of the APC promoter, the MethyLight assay was used to detect the CpG island methylation level of the APC promoter in 57 pairs of HCC and matched non-tumor liver tissues. The data indicated that the levels of the APC promoter were higher in 45 out of 57 (78.95%) tumor tissues compared with adjacent non-cancerous liver tissues (Fig. 1A). The rate of DNA methylation [(gene sample/Alu-C4 sample)/(gene SssI-treated sample/Alu-C4 SssI-treated sample) × 100] in the APC gene promoter was also revealed to be significantly higher in the HCC tissues compared with the adjacent non-cancerous tissues (paired t-test, P=0.0003; Fig. 1B). The median rate of methylation in the HCC tissues was 9.93% (range, 0.02–150.8) and the median rate in the non-cancerous liver tissues was 2.2% (range, 0.0035–56.84). The median rate of DNA methylation was upregulated by 4.51-fold in the HCC tissues compared with the non-cancerous tissues. Occasionally PMR values may be >100%; this occurred in cases when the M.SssI treatment of the standard DNA was not complete or in cases of aneuploidy of the gene locus of interest. Details are shown in Table I. Overall, these data showed that the rate of DNA methylation in the APC locus was upregulated in the HCC tissue compared with the non-cancerous adjacent tissues.

### Correlation between clinicopathological data and methylation level of the APC promoter in HCC

**Figure 2:**
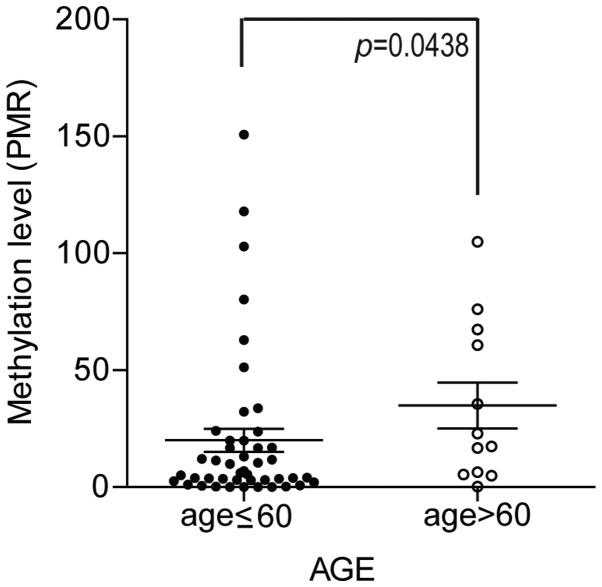
Methylation level of the APC promoter in the HCC samples is correlated with patient age. PMR, PMR, percentage of methylation; APC, adenomatous polyposis coli; HCC, hepatocellular carcinoma.

To determine whether hypermethylation of APC can be a characteristic biomarker of certain types of HCC, the correlations between the methylation level of the APC promoter in the HCC samples and the clinicopathological parameters were analyzed. Notably, the methylation level of the APC promoter in the HCC samples was higher in older patients when the cut-off was set at 60 years old. In the 12 patients who were >60 years old, the mean methylation level was 34.90%, while in the 45 patients who were <60 years old, the mean methylation level was 20.04% (P=0.0438; Fig. 2).

**Figure 3:**
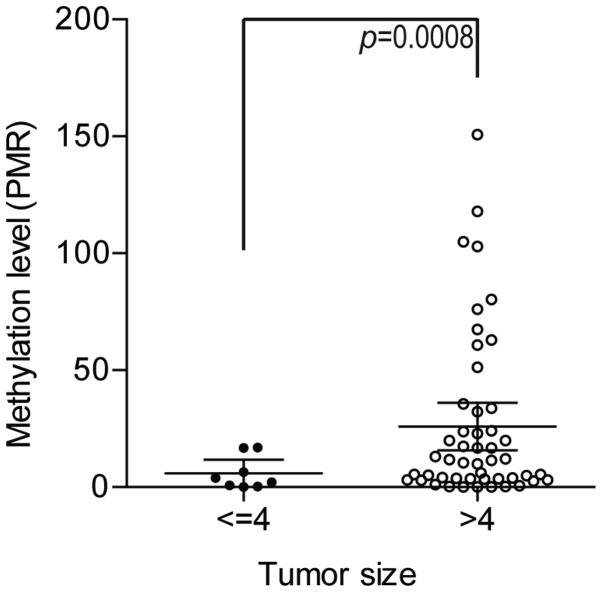
Methylation level of the APC promoter in the HCC samples is correlated with tumor size. PMR, PMR, percentage of methylation; APC, adenomatous polyposis coli; HCC, hepatocellular carcinoma.

Additionally, the CpG island methylation level of the APC promoter in the HCC samples was significantly higher in the patients with larger tumors when the cut-off was set at 4 cm. Of the 8 tumors with a size of >4 cm, the mean methylation level of the tumor suppressor gene APC promoter was 13.06%, which is significantly higher than the mean methylation level of the remaining 49 patients (2.96%) (P=0.0008; Fig. 3). When correlated with other clinical parameters, including gender, presence of a tumor embolus or tumor capsule, tumor-node-metastasis stage, paracirrhosis and α-fetoprotein level, there was no significant correlation with the methylation level of the APC promoter in the HCC samples (Table I).

### Correlation between survival and methylation level of the APC promoter in HCC

**Figure 4:**
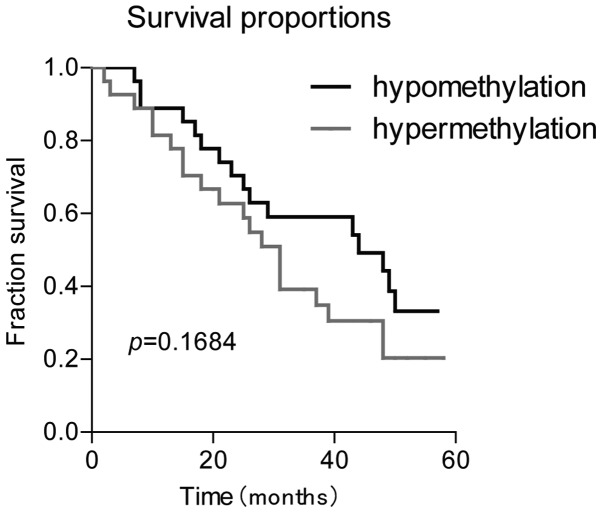
Hypermethylation of the APC gene promoter did not affect the overall survival rate of the HCC patients. APC, adenomatous polyposis coli; HCC, hepatocellular carcinoma.

The survival rate of the HCC patients was also explored and the association with the methylation status of the APC promoter was evaluated. Complete follow-up data were available in 54/57 patients (94.74%). Of the 54 patients, 36 (66.67%) succumbed to disease (median follow-up, 50.5 months) and 18 (33.33%) survived (median, 24 months). Overall, the patients survived between 2 and 58 months, with a median of 30 months. The methylation rate in HCC tissues above the mean PMR were defined as hypermethylation cases and HCC tissues with methylation rates below the mean PMR were defined as hypomethylation cases. The follow-up results did not reveal any significant difference between the overall survival rate in the hypomethylation or hypermethylation cases (P=0.1684; Fig. 4).

## Discussion

Promoter hypermethylation of tumor suppressor or tumor-related genes plays a significant role in tumorigenesis. Hypermethylation of APC, a well-characterized tumor suppressor, has been detected frequently in HCC in various studies (2–12). However, the majority of previous studies used methylation-specific PCR, which has been considered as an easy-to-use qualitative method for the detection of DNA methylation. DNA hypermethylation can be assessed by methylation-specific PCR, which although extremely sensitive, is dependent on the number of PCR cycles, the mixture conditions and the amount of DNA (16). Therefore, the reported methylation frequencies of identical genes in HCC varies between studies (2,4–12). Taqman probe-based quantitative methylation-specific PCR (MethyLight) can overcome issues associated with PCR cycling and provide reliable quantitative information about the methylation status of the target CpG island loci (11).

The findings of the present study showed that the promoter region of the APC gene was methylated in 35.09% of the non-cancerous liver tissues and 64.91% of HCC samples using a cut-off set at 4% PMR, which indicates a high methylation frequency of the APC gene promoter in HCC and a moderate methylation frequency in the paired non-tumorous liver tissues. The moderate methylation frequencies observed in the non-tumorous liver tissue indicate epigenetic evidence for field cancerization involved in the early stage of liver carcinogenesis (5). The fact that the methylation frequency of the APC promoter in the HCC samples was much higher than that in the paired non-tumorous liver tissues indicates that this aberrant methylation alteration is a quantitative epigenetic change that accumulated through the process of hepatocarcinogenesis. The results of the present study were highly consistent with those of the previously published studies, regardless of the detection method used (2,4–12).

Aberrant methylated genes can be used as biomarkers for tumor classification and early detection, and to detect the response to treatments, including traditional chemotherapy drugs, target therapy and epigenetic agents (14). In the present study, the CpG island methylation level of the APC promoter in the HCC samples was significantly higher in the patients with larger tumors when the cut-off was set at 4 cm. Of the 8 tumors with a size of >4 cm, the mean methylation level of the tumor suppressor gene APC promoter was 13.06%, while for the remaining 49 patients with a smaller tumor, the mean methylation level was 2.96%. This result may be due to the inactivity of the APC protein. The functionally inactive APC protein, created by transcriptional silencing through promoter methylation, inhibits interactions with β-catenin. This allows the direct interaction of β-catenin with the lymphoid enhancer factor-T cell factor family of transcription factors and the promotion of cells into the cell cycle (3).

In the present study, the methylation level of the APC promoter in the HCC samples was also identified to be higher in older patients when the cut-off was set at 60 years old. In the 12 patients who were >60 years old, the mean methylation level was 34.90%, while in the 45 patients who were <60 years old, the mean methylation level was 20.04%. This is the first APC hypermethylation study that has shown a correlation between methylation level and patient age. No similar result has been reported in previous studies. However, the methylation level of another frequently detected tumor suppressor gene, Ras association domain family 1A (RASSF1A), has been reported to be correlated with patient age. A study by Di Gioia *et al* demonstrated that the age-related methylation of the RASSF1A promoter takes place early in a small subpopulation of cells of the human liver (17). Conversely, Feng *et al* and Zhong *et al* indicated that no association was apparent between the methylation of the RASSF1A gene promoter and patient age (18,19). Demonstrations of the association between the methylation level of the APC promoter and patient age are further required by other quantitative methylation detection methods, including pyrosequencing, in prospective studies of various geographical cohorts.

Last, but not least, the correlation between APC hypermethylation and survival rate was analyzed in the present study by univariate analysis using KM and log-rank tests. The aberrant hypermethylation of the APC promoter had no significant impact on the overall survival rate. Thus the methylation level of the APC promoter in HCC may not serve as a promising prognostic biomarker for the disease. This result was inconsistent with a previous study in which elevated plasma methylation levels of APC were associated with a poorer overall survival rate (20).

In summary, using a highly precise and quantitative tool for detecting epigenetic changes in clinical samples of HCC and corresponding non-cancerous liver tissues, the present study identified that the methylation level of the APC promoter in HCC tissues was significantly higher than in paracancerous liver tissues. This result indicated that hypermethylation of the APC promoter is an early event in hepatocarcinogenesis and is quantitatively accumulated in the development of HCC. In addition, the methylation level of the APC promoter is correlated with the age at the time of HCC diagnosis and the tumor size. However, a higher degree of APC promoter methylation in tumor tissues does not appear to be responsible for a poorer overall survival rate, as has been reported in previous studies. Therefore, the methylation level of the APC promoter in HCC may not serve as a good prognostic biomarker of HCC. This result requires further elucidation by other quantitative methylation detection methods, including pyrosequencing, in prospective studies of various geographical cohorts.

## Figures and Tables

**Table I:** Clinical information of 57 paired HCC samples and the corresponding APC promoter methylation level detected by the MethyLight assay.

Parameters	Number of cases	PMR of NT (mean ± SEM)	PMR of HCC (mean ± SEM)	P-value
Total	57	3.09±0.684	11.64±2.236	0.0003^*^
Age, years				
>60	12	9.62±4.581	34.90±9.879	0.0438^*^
≤60	45	5.22±1.221	20.04±4.934	
Gender				
Male	49	6.88±1.558	23.39±4.842	0.9000
Female	8	1.64±0.551	21.76±11.97	
Tumor size, cm				
>4	8	6.85±4.129	2.955±1.256	0.0008^*^
≤4	49	6.03±0.451	13.06±2.539	
Tumor embolus^a^				
Positive	21	7.20±2.376	26.50±6.616	0.3305
Negative	35	5.43±1.713	21.68±6.091	
Tumor capsule^c^				
Complete	15	7.69±4.081	26.68±9.332	0.8195
Incomplete	22	6.09±0.698	29.67±8.660	
TNM stage^a^				
I, II	34	4.77±1.725	12.84±3.382	0.5750
III, IV	22	8.13±2.285	10.20±2.523	
Paracirrhosis^a^				
Positive	38	4.74±1.165	28.22±6.133	0.1295
Negative	18	8.94±3.514	13.51±4.889	
AFP, μg/l^b^				
<20	13	7.34±3.072	23.57±8.308	0.7902
20–400	25	7.30±2.584	25.25±8.272	
>400	17	3.16±0.746	18.05±5.244	

Information was missing from ^a^one, ^b^two and ^c^20 samples. AFP, α-fetoprotein; TNM, tumor-node-metastasis; PMR, percentage of methylation; SEM, standard error of the mean; NT, non-tumor tissue; HCC, hepatocellular carcinoma; APC, adenomatous polyposis coli.
